# A Single Immunization with Soluble Recombinant Trimeric Hemagglutinin Protects Chickens against Highly Pathogenic Avian Influenza Virus H5N1

**DOI:** 10.1371/journal.pone.0010645

**Published:** 2010-05-14

**Authors:** Lisette A. H. M. Cornelissen, Robert P. de Vries, Els A. de Boer-Luijtze, Alan Rigter, Peter J. M. Rottier, Cornelis A. M. de Haan

**Affiliations:** 1 Central Veterinary Institute of Wageningen University, Lelystad, The Netherlands; 2 Virology Division, Department of Infectious Diseases & Immunology, Faculty of Veterinary Medicine, Utrecht University, Utrecht, The Netherlands; University of Georgia, United States of America

## Abstract

**Background:**

The highly pathogenic avian influenza (HPAI) virus H5N1 causes multi-organ disease and death in poultry, resulting in significant economic losses in the poultry industry. In addition, it poses a major public health threat as it can be transmitted directly from infected poultry to humans with very high (60%) mortality rate. Effective vaccination against HPAI H5N1 would protect commercial poultry and would thus provide an important control measure by reducing the likelihood of bird-to-bird and bird-to-human transmission.

**Methodology/Principal Findings:**

In the present study we evaluated the vaccine potential of recombinant soluble trimeric subtype 5 hemagglutinin (sH5^3^) produced in mammalian cells. The secreted, purified sH5^3^ was biologically active as demonstrated by its binding to ligands in a sialic acid-dependent manner. It was shown to protect chickens, in a dose-dependent manner, against a lethal challenge with H5N1 after a single vaccination. Protected animals did not shed challenge virus as determined by a quantitative RT-PCR on RNA isolated from trachea and cloaca swabs. Also in mice, vaccination with sH5^3^ provided complete protection against challenge with HPAI H5N1.

**Conclusions/Significance:**

Our results demonstrate that sH5^3^ constitutes an attractive vaccine antigen for protection of chickens and mammals against HPAI H5N1. As these recombinant soluble hemagglutinin preparations can be produced with high yields and with relatively short lead time, they enable a rapid response to circulating and potentially pandemic influenza viruses.

## Introduction

Influenza A viruses are enveloped, negative-strand RNA viruses with a segmented genome. They infect a large variety of animal species, although birds are considered to constitute the reservoir from which all influenza A viruses in other species originate [1], [2]. On the basis of the antigenic properties of their two surface glycoproteins, hemagglutinin (HA) and neuraminidase (NA), influenza A viruses are classified into 16 HA (H1–16) and 9 NA (N1–9) subtypes. The highly pathogenic avian influenza (HPAI) virus H5N1 causes multi-organ disease and death in poultry, resulting in significant economic losses in the poultry industry. HPAI H5N1 also poses a major public health threat as it can be transmitted directly from infected poultry to humans with very high (60%) mortality rate. It is widely accepted that continued human exposure to influenza viruses circulating in wild and domestic avian species poses a permanent pandemic threat [3], [4]. Effective vaccination against HPAI H5N1 would protect commercial poultry and would thus provide an important control measure by reducing the likelihood of bird-to-bird as well as bird-to-human transmission. Therefore, the development of efficacious influenza vaccines is of high veterinary and public health importance.

Conventional vaccine preparations against HPAI H5N1 are produced by propagating virus in embryonated chicken eggs or mammalian cells. The vaccine viruses used are either reassortants or low pathogenic wild-type avian viruses. Either way, the viruses need to be selected for their ability to multiply in eggs or cultured cells, which may preclude the best genetic match to the circulating HPAI H5N1 strains. In addition, these vaccines have several other limitations as reviewed elsewhere [5], [6]. Hence, other vaccine strategies against HPAI H5N1 have been explored including the use of, among others, live attenuated influenza vaccines [7], [8], live vaccines based on heterologous viral vectors such as pox virus [9], adenovirus [10], baculovirus [11] and Newcastle disease virus [12], and DNA vaccination [13]. While these different strategies often showed promising results, their applicability ultimately will depend on various important issues including safety, efficacy, production and costs [5].

Protective immunity against influenza virus infection and disease is primarily conferred through HA via the induction of anti-HA antibodies. As protection from influenza virus infection correlates with anti-HA titers, nearly all vaccine approaches aim to induce high levels thereof. Therefore, the HA protein is the antigen of choice for the development of recombinant subunit vaccines to protect against HPAI H5N1. An influenza vaccine based on recombinant purified HA could offer the following advantages: I) The HA antigen can be produced using safe, quality-controlled and scalable conditions. II) There will be no need for virus cultivation, thus avoiding the necessity a) to obtain viruses that replicate efficiently in eggs or cell culture, b) to use biocontainment facilities and c) to inactivate the virus using procedures that may affect antigenicity and raise safety concerns. III) The recombinant HA protein can be highly purified thereby limiting adverse reactions caused e.g. by the presence of egg contaminants. IV) Immunization with recombinant HA will allow the serological differentiation of naturally infected from vaccinated animals/flocks (the so-called DIVA principle; [14]). IV) Recombinant HA vaccines are manufactured with a relatively short lead time, allowing an accelerated response to emerging influenza strains.

Recombinant HA can be produced using different expression systems. When expressed in *E. coli* the resulting HA protein gave rise to the induction of hemagglutination inhibition (HI) titers upon immunization of mice [15]. However, as proper folding and trimerization of the HA protein requires multiple posttranslational modifications including glycosylation and disulfide bond formation, expression of the HA protein in higher eukaryotic systems is likely to result in superior antigenicity. Thus, mammalian cell-derived HA trimers were found to induce much higher levels of neutralizing antibodies than similarly produced monomeric HA protein [16]. The baculovirus expression system has been used to produce strain specific HA antigens in insect cells, which were shown to protect against a HPAI H5N1 challenge [17]. Such full-size HA proteins may, however, be limited in their efficacy because the membrane proteins may not retain their native membrane-bound structure upon purification [18]. Hence, the production of recombinant, soluble, stable HA trimers that are secreted from the cells seems like an attractive alternative approach. Such HA trimers, expressed either in insect or mammalian cells, were indeed shown to elicit neutralizing antibodies [16] and to partially protect mice against HPAI H5N1 challenge infection [19].

In view of their promising potential we have evaluated recombinant soluble HA trimers in chickens and mice for their ability to induce protective immunity against infection with HPAI H5N1. To this end, recombinant soluble H5 proteins provided with a GCN4 trimerization motif and a STREP-tag II, the latter for purification purposes, were expressed in mammalian cells. The recombinant soluble H5 trimers (sH5^3^) were purified from the culture supernatants using a simple one-step purification protocol and characterized with respect to their oligomeric state and bioactivity. Subsequently, vaccination with the sH5^3^ preparation was shown to provide complete protection against challenge with HPAI H5N1 both in mice and in chickens, in the latter already after a single immunization.

## Materials and Methods

### Genes and expression vectors

A cDNA clone corresponding to residues 18 to 523 (H3 numbering) of the HA from A/Viet Nam/1203/2004 (H5N1) (Genbank accession no. ABW90137.1) was synthesized using human-preferred codons by GenScript USA Inc. In this clone the predicted HA ectodomain protein lacks a multibasic cleavage site. The cDNA was cloned into the pCD5 expression vector for efficient expression in mammalian cells [20]. The pCD5 vector had been modified such that the HA-encoding cDNA was cloned in frame with DNA sequences coding for a signal sequence, an artificial GCN4 isoleucine zipper trimerization motif (KRMKQIEDKIEEIESKQKKIENEIARIKK) [21] and the Strep-tag II (WSHPQFEK; IBA, Germany). The resulting vector encodes the soluble trimeric H5 protein designated as sH5^3^.

### Protein expression and purification

pCD5 expression vectors containing the HA ectodomain-encoding sequences were transfected into HEK293S GnTI(−)cells [22] using polyethyleneimine I (PEI) in a 1∶5 ratio (µg DNA∶ µg PEI). At 6 h post transfection, the transfection mixture was replaced by 293 SFM II expression medium (Invitrogen), supplemented with sodium bicarbonate (3.7 g/liter), glucose (2.0 g/liter), Primatone RL-UF (3.0 g/liter), penicillin (100 units/ml), Streptomycin (100 µg/ml), glutaMAX (Gibco), and 1,5% dimethyl sulfoxide. Tissue culture supernatants were harvested 5–6 days post transfection. HA protein expression and secretion was confirmed by sodium dodecylsulfate (SDS)-polyacrylamide gel electrophoresis (PAGE) followed by western blotting using a mouse anti-Strep-tag antibody (IBA, Germany). HA proteins were purified using Strep-tactin Sepharose beads according to the manufacturer's instructions (IBA, Germany). The concentration of purified protein was determined by using a Nanodrop 1000 spectrophotometer (Isogen Life Sciences) according to the manufacturer's instructions.

### Biological characterization of recombinant HA

The oligomerization status of the sH5^3^ proteins was determined by analyzing the elution profile using a Superdex200GL 10–300 column (GE Healthcare). Sialic acid-binding activity of sH5^3^ was assessed using a fetuin solid phase assay. 1 µg/ml fetuin per well was used to coat 96-well Nunc MaxiSorp plates. When indicated in the figure legend, fetuin was treated with *Vibrio Cholera* derived neuraminidase (VCNA; Roche) followed by three washing steps. sH5^3^ was pre-complexed with horseradish peroxidase (HRP)-linked anti-Strep-tag antibody (2∶1 molar ratio) for 30 min at 0°C prior to incubation of limiting dilutions on the fetuin-coated plates (60 min, room temperature [RT]). HA-binding was subsequently detected using tetramethylbenzidine substrate (BioFX) in an ELISA reader (EL-808 [BioTEK]), reading the OD at 450 nm.

### Vaccination-challenge experiments in chickens

Animal studies were conducted at the Central Veterinary Institute (CVI), Lelystad, under BSL3 conditions and after approval by the Animal Ethic Committee. In the first chicken experiment, 6 weeks old SPF white Leghorn chickens (Lohmann, Cuxhaven, Germany) were used (10 chickens per group). One group of animals was immunized twice (on day 0 and 21) by intramuscular (i.m.) injection (0.5 ml) of 10 µg sH5^3^ adjuvanted with Stimune as recommended by the manufacturer [Prionics, Lelystad]). As a challenge control, the other group received an equal volume of PBS in Stimune (mock vaccinated). Three weeks after the vaccination (day 42), the birds were challenged by inoculation with 10^5^ median tissue culture infective dose (TCID_50_) of A/Viet Nam/1194/04 virus (0.1 ml intranasally [i.n.] and 0.1 ml intratracheally [i.t.]) and monitored over a period of 14 days for signs of disease. Blood samples were collected at 3 weeks after each immunization (on day 21 and 42 post infection [p.i.]).

For the next experiment, 70 one-day-old layer hens (white Leghorn) were purchased from a local breeder. The chickens had been vaccinated against Newcastle disease virus and infectious bronchitis virus at the age of one day according to the farm's routine and were raised in CVI's animal facility. At the age of 6 weeks, the birds were transported to the BSL-3 facility and allocated to 7 experimental groups of 10 birds each. The animals of six groups were immunized once (on day 21) or twice (on day 0 and 21) by i.m. injection of 10, 2 or 0.4 µg sH5^3^ antigen adjuvanted with Stimune. When immunized twice, the same doses were given on day 0 and 21. As a challenge control, one group was mock-vaccinated twice (on day 0 and 21) with PBS in Stimune. Four weeks after vaccination (day 49), blood samples were taken and the chickens were challenged as above and observed daily for clinical signs during 14 days. Trachea and cloaca swabs were taken from each chicken on day 2, 4 and 7 p.i.

### Vaccination-challenge experiment in mice

Female, specified pathogen-free (SPF) 9-week-old BALB/c mice (Charles River Laboratories; 10 animals per group) were immunized once (on day 21) or twice (on day 0 and 21) by i.m. injection (0.2 ml) of 2 µg sH5^3^ adjuvanted with Stimune. As a challenge control, one group of mice was mock vaccinated. Three weeks after the vaccination, mice were anaesthetized with ketamin/xylazin by intraperitoneal injection and inoculated intranasally with 50 µl of H5N1A/Viet nam/1194/04 containing ∼10 median lethal dose (LD_50_; 3.7 ^10^log TCID_50_; provided by Dr. Alan Hay from the WHO Influenza Centre at the National Institute for Medical Research, London). The mice were weighed daily and examined for signs of illness during the next 14 days. Clinical signs were recorded using a scoring system (0 = no clinical signs; 1 = rough coat; 2 = rough coat, less reactive, passive during handling; 3 = rough coat, rolled up, laboured breathing, passive during handling; 4 = rough coat, rolled up, laboured breathing, unresponsive). Animals reaching a score of 4 were euthanized. Surviving animals were bled and sacrificed on day 14 p.i.

### Virus detection assay

Trachea and cloaca swabs were stored in cold tryptose phosphate broth supplemented with antibiotics. The medium was clarified by low-speed centrifugation and the supernatant was harvested, aliquoted and stored at −70°C. Upon thawing, trachea and cloaca swabs sampled from the same bird on the same day were pooled and the viral RNA was extracted from 200 µl using a MagNA Pure LC Total Nucleic Acid Isolation Kit (Roche). Subsequently, cDNA was synthesized using reverse primer 5′-CACTGGGCACGGTGAGC-3′ and part of the M1 gene was amplified by running 45 cycles of Light Cycler PCR using primer 5′-CTTCTAACCGAGGTCGAAACGTA-3′ as the reverse primer in the presence of the TaqMan fluorescent probe 5′-6FAM-TCAGGCCCCCTCAAAGCCGA-X-ph. Negative and positive control samples were tested in parallel. The lower limit of detection was determined to be approximately 500 TCID_50_. Some samples gave inconclusive results, meaning that they gave only a very weak signal (fluorescence<0.07) after >31 cycles.

### Hemagglutination inhibition (HI) assay

Heat-inactivated immune sera from chicken blood samples were tested for hemagglutination inhibition (HI) activity with 1% chicken red blood cells and 4 hemagglutinating units (HAU) of H5N1 (A/Viet Nam/1194/04 NIBRG-14). In addition, the chicken sera were tested for HI activity using 8 HAU (67 ng) of recombinant soluble trimeric HA protein. To this end the recombinant proteins were precomplexed with the anti-Strep-tag antibody as described above, mixed with limiting dilutions of the chicken sera and incubated with 0.5% chicken red blood cells. Red button formation was scored as evidence of hemagglutination. Antibody titers were expressed as the reciprocal of the highest serum dilution showing HI. Immune sera prepared from mouse blood samples were treated with VCNA, heat-inactivated at 56°C for 30 min and tested by HI assay using 8 HAU of sH5^3^ as described above.

### ELISA

Total antibody titers against sH5^3^ were determined by using a sH5^3^-specific ELISA. To this end, 96-well Nunc MaxiSorp plates coated with 0.5 µg sH5^3^ per well were incubated with limiting dilutions of chicken or mouse sera. After extensive washing, the plates were incubated with goat-anti-chicken or goat-anti-mouse antibodies conjugated with HRP. Peroxidase activity was visualized using tetramethylbenzidine substrate (BioFX) and an ELISA reader (EL-808 [BioTEK]), reading the OD at 450 nm. The OD values of the 250-fold diluted samples, which were in the logarithmic phase of the curve, were plotted.

## Results

### Expression, purification, and characterization of sH5^3^

**Figure 1:**
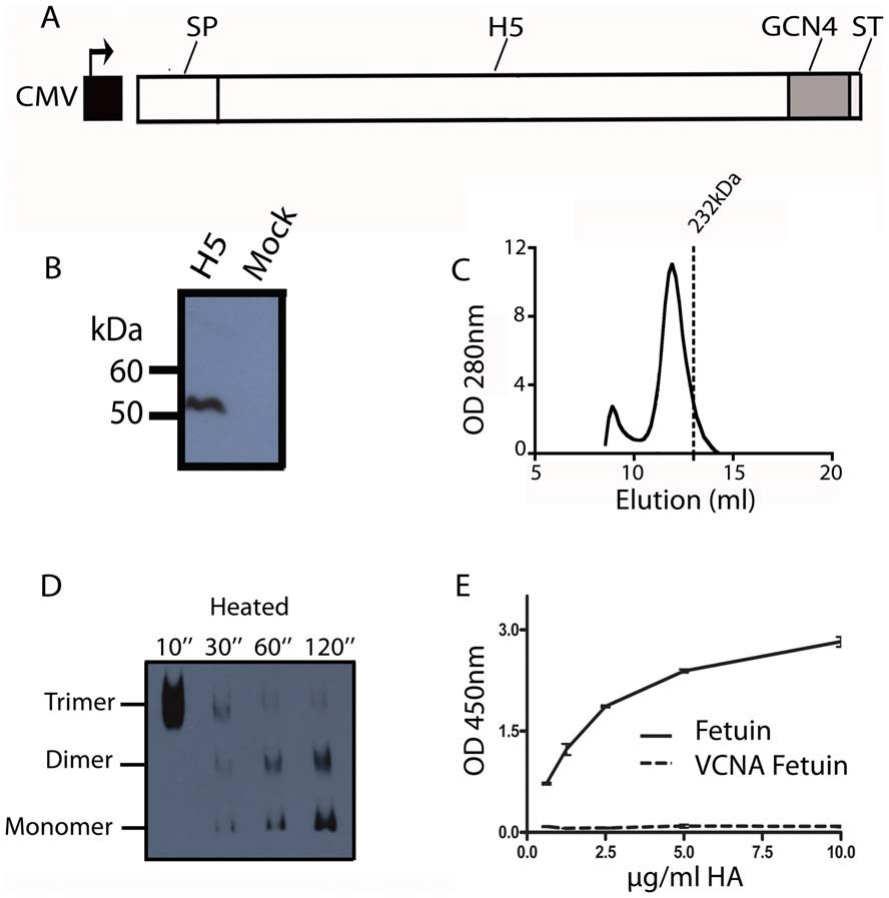
Expression, purification and biological activity of recombinant, soluble trimeric H5 protein. (A) Schematic representation of the H5 expression cassettes used. The H5 ectodomain encoding sequence (H5) was cloned in frame with DNA sequences coding for a signal peptide (SP), the GCN4 isoleucine zipper trimerization motif (GCN4) and the Strep-tag II (ST) under the control of a CMV promoter. (B) H5 expression and secretion into the culture media was analyzed by SDS-PAGE followed by western blotting. The recombinant protein was detected using a mouse anti-Strep-tag antibody. (C) Analysis of purified recombinant H5 proteins by gel filtration. Shown is the elution profile of a H5 protein preparation using a Superdex200GL 10–300 column. The elution of a 232 kDa catalase control is indicated by the line. (D) Blue native-PAGE analysis of the recombinant H5 protein. The position in the gel of the momomeric, dimeric and trimeric ectodomain species observed after heating of the HA sample prior to electrophoresis is indicated. (E) Recombinant soluble H5 trimers were complexed with a HRP-conjugated mouse antibody directed against the Strep-tag prior to their application in a fetuin binding assay. HA binding was also assessed after treatment of fetuin with VCNA (fetuin+VCNA).

In order to express soluble trimeric subtype 5 HA (sH5^3^) in mammalian cells, the H5 ectodomain-coding sequence was first cloned into an appropriate expression vector. In the pCD5 vector used, the H5-sequence was preceded by a signal peptide-encoding sequence, to allow efficient secretion of the recombinant protein, and followed by sequences coding for the GCN4 isoleucine-zipper trimerizaton motif [21] and the Strep-tag II, the latter for purification purposes (Fig. 1A). Expression of the H5 ectodomain was achieved by transient transfection of the expression plasmid into HEK cells. Expression and secretion of the H5 protein was verified by subjecting cell culture supernatant to gel electrophoresis followed by western blotting using an antibody directed against Strep-tag II (Fig. 1B). The results show that the recombinant H5 protein could be readily detected in the cell culture supernatant after transfection of the cells with the expression plasmid, but not after mock transfection. The secreted H5 protein was purified in a single step protocol by using Strep-tactin sepharose beads. Protein yields varied between 0.4–1 mg of recombinant protein per 100 ml cell culture medium. After purification of the H5 protein from the cell culture supernatant, the oligomeric state of the H5 protein was analyzed by gel filtration column chromatography (Fig. 1C). The bulk of the H5 protein eluted with the velocity of an oligomer, while only a minor fraction was found as aggregates in the void volume. The trimeric nature of the H5 oligomer was confirmed using blue-native gel electrophoresis followed by western blotting (Fig. 1D). When the H5 preparation was heat-denatured for increasing time periods prior to electrophoresis, the initially trimeric HA species dissociated into dimers and monomers. The biological activity of the purified sH5^3^ was studied using a solid phase-binding assay with the sialylated blood glycoprotein fetuin. Binding of sH5^3^ was measured by means of the HRP conjugated to the anti-Strep-tag II antibody as detailed in the Material and Methods section. The H5 preparation exhibited a concentration dependent binding to the fetuin. This binding was sialic acid-dependent, as no binding was observed when the fetuin had been treated with neuraminidase (VCNA; Fig. 1E). In conclusion, biologically active sH5^3^ was efficiently produced using a mammalian expression system and readily purified.

### Efficacy of sH5^3^ as a vaccine against lethal HPAI H5N1 infection in chickens

**Figure 2:**
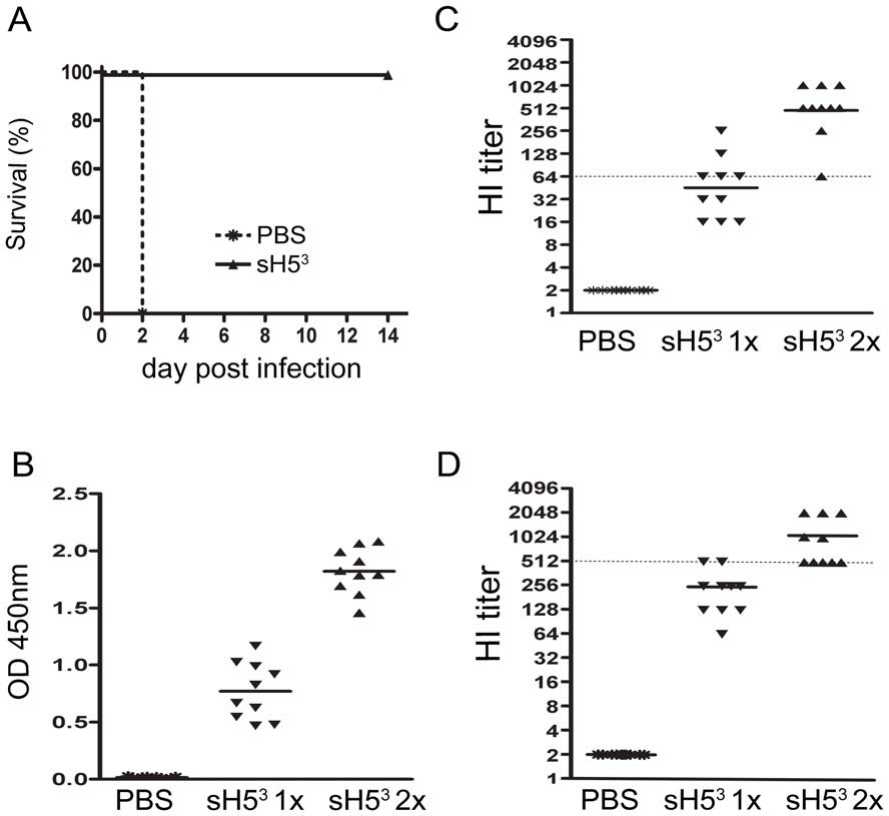
sH5^3^ vaccination in chickens. Ten chickens were immunized two times with 10 ug sH5^3^ (on day 0 and 21). As a challenge control, chickens were mock-treated (PBS). Three weeks after the vaccination, all birds were challenged with 10^5^ TCID_50_ of HPAI H5N1 A/Viet Nam/1194/04 (A) Kaplan-Meier survival curves, indicating percentage mortality on each day for each group (B–C–D) Blood samples were collected 3 weeks after the first immunization (sH5^3^ 1×) and 3 weeks after the second vaccination (sH5^3^ 2×). The sH5^3^ antibody levels as determined by ELISA (B), the HI titers against H5N1 (NIBRG-14) (C) and the HI titers against sH5^3^ (D) in serum for each bird. Bars represent geometric means per group. The dotted lines indicate the lowest antibody levels correlating with protection in this experiment.

To examine the immunogenicity of sH5^3^ and its potential as a vaccine a first experiment in chickens was performed, in which 10 chickens were vaccinated twice (on day 0 and 21) i.m. with 10 µg of Stimune-adjuvanted sH5^3^ and challenged 3 weeks later by i.n./i.t. inoculation of a lethal dose of A/Viet Nam/1194/04 virus. Another 10 birds were mock-vaccinated to serve as challenge controls. As shown in Fig. 2, the boost vaccination with 10 µg sH5^3^ conferred complete protection. None of the vaccinated chickens died or showed symptoms indicative of influenza-related disease, whereas all mock-vaccinated chickens succumbed within 2 days. Serological analysis showed that none of the mock-vaccinated animals contained antibodies against sH5^3^ as determined by a sH5^3^-specific ELISA (Fig. 2B). In contrast, all immunized animals demonstrated appreciable levels of HA antibodies already after a single immunization and these levels increased further after the boost. These total antibody levels against sH5^3^ correlated nicely with the HI titers against H5N1 (Fig. 2C). All mock-vaccinated chickens had a HI titer below the detection limit. HI antibody titers observed after the sH5^3^ boost reached with a maximum of 1024 and a minimum of 64, which was apparently still sufficient to protect the animal against the lethal challenge. Interestingly, 50% of the birds developed HI antibody titers equal to 64 or higher already three weeks after the first vaccination. In addition, HI titers were also determined by using sH5^3^ rather than H5N1 virus as the hemagglutinating agent, which gave essentially similar results (compare Fig. 2D and 2C), demonstrating the reliability of the assay. In summary, the results show that chickens vaccinated twice with sH5^3^ are protected against a lethal challenge with H5N1. The HI titers observed suggested that one vaccination might already be sufficient to confer protection against HPAI H5N1.

### Vaccination efficacy in chickens: effect of antigen dose and vaccination booster

**Figure 3:**
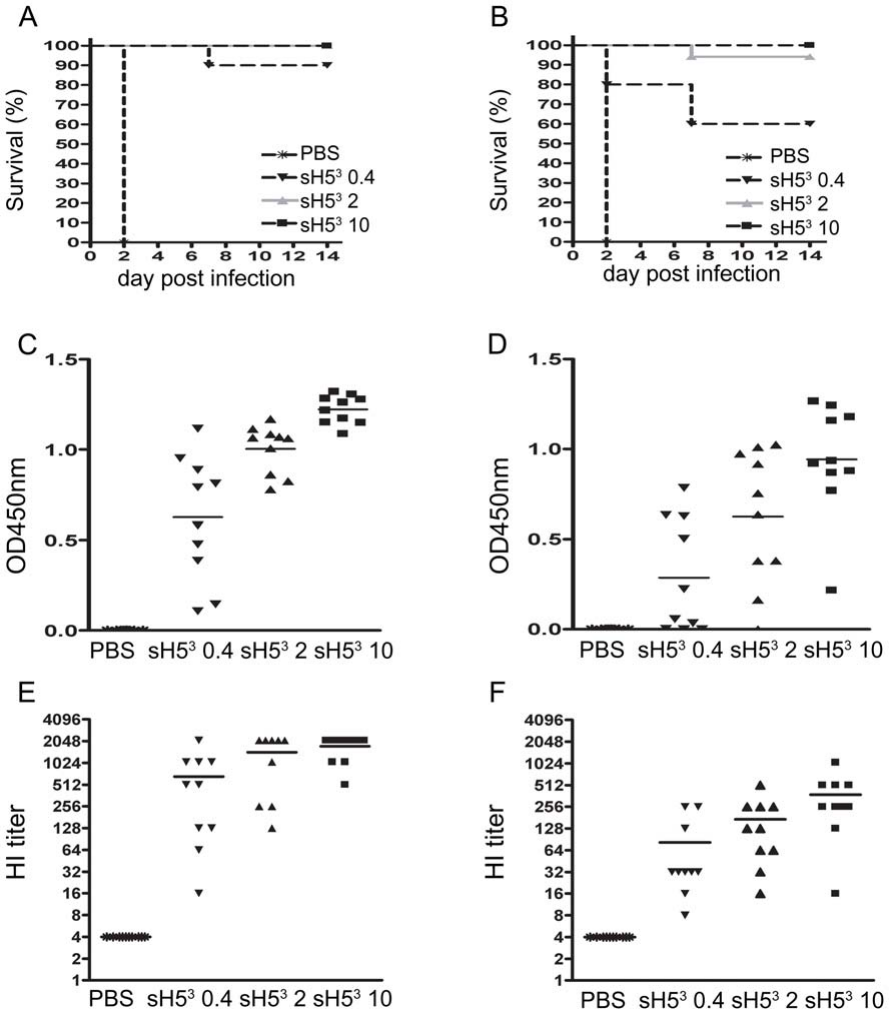
sH5^3^ dose titration after a single or boost vaccination in chickens. Seven groups of 10 chickens were immunized i.m. with 10, 2 or 0.4 µg sH5^3^ either once or twice with 3 weeks interval. As a challenge control, one group was mock-treated (PBS). Four weeks after the vaccination, all chickens were challenged with 10^5^ TCID_50_ of HPAI H5N1 A/Viet Nam/1194/04. Kaplan-Meier survival curves, indicating percentage mortality on each day for each group that was (mock-)vaccinated twice (A) or once (B). (C–D) The sH5^3^ antibody levels at the day of challenge as determined by ELISA for each chicken that was (mock-)vaccinated twice (C) or once (D). (E–F) Serum HI titers in the same sera, measured against sH5^3^ for the chickens that were (mock-)vaccinated twice (E) or once (F). Bars represent the geometric means for the test groups.

These results prompted us to determine the minimal sH5^3^ dose required to confer protection and to examine whether a single dose could already be sufficient. In the second vaccination-challenge experiment, chickens were thus vaccinated with 10, 2 or 0.4 µg of sH5^3^ either once or twice and challenged four weeks later by infection with a lethal dose of A/Viet Nam/1194/04 as described above. The results are shown in Fig. 3. Again, all mock-vaccinated birds succumbed to the infection within 2 days. Vaccinating twice with a dose of 0.4 µg of sH5^3^ was sufficient to protect 90% of the chickens against mortality, while all chickens survived when a dose of 2 or 10 µg was similarly administered (Fig. 3A). Interestingly, also single vaccination with sH5^3^ could induce sufficient immunity to protect chickens against lethal infection (Fig. 3B); when a dose of 2 µg was given only one chicken died (90% protection), whereas a dose of 10 µg was protective to all birds. Even after a single dose of 0.4 µg, 60% of the chickens were protected against death.

Serological analysis showed that protection against the lethal H5N1 challenge correlated well with the observed antibody levels against sH5^3^ as determined by ELISA (Fig. 3C–D) and by HI assay (Fig. 3E–F). Both assays revealed a dose-dependent antibody response, which was substantially enhanced after the booster immunization. Relatively high HA antibody levels were observed after two immunizations, even with the lower dose, except in two animals, one of which did not survive the challenge (Fig. 3C). Also after a single immunization, significant antibody levels were measured, except again in the low dose group. Here, 5 animals had hardly measurable ELISA titers. Consistently, 4 of these animals succumbed to the challenge. Also the one animal that died after a single immunization with 2 µg of sH5^3^ did not have detectable sH5^3^ antibodies. Essentially the same results as with the ELISA were obtained with the HI assay using sH5^3^ as the hemagglutinating agent (Fig. 3E–F). Thus, the animals that succumbed to the lethal challenge after a single immunization also exhibited the lowest HI titers.

### Shedding of challenge virus from vaccinated chickens

We analyzed whether vaccination with sH5^3^ decreased or prevented chickens from shedding challenge virus. For practical reasons, virus shedding was analyzed by a quantitative RT PCR rather than by measuring infectious virus titers. To this end, trachea and cloaca swabs were taken from the chickens of the vaccine dose titration experiment of Fig. 3 at 2, 4 and 7 days after the challenge inoculation. The tracheal and the cloacal swab sampled from each chicken at each particular day were pooled and the presence of viral RNA in the pooled swabs was analyzed using a quantitative RT PCR assay detecting the M1 gene. The results are shown in Table 1. Of the chickens that received a booster vaccination, only 2 birds, both of which had received the lowest amount of antigen, tested positive. Notably, these were the two animals that developed the lowest sH5^3^-specific antibody titers (Fig. 3C). One of these animals did not survive the challenge. Virus shedding could not be detected in any of the other birds, although 3 swabs gave inconclusive results. Of the chickens vaccinated only once, all animals that died tested positive. None of the birds vaccinated with 10 µg sH5^3^ tested positive. Of the chickens vaccinated with a lower dose and surviving, two tested positive, but only at day 2 p.i. In conclusion, the vaccinated birds that could control the lethal HPAI H5N1 challenge infection exhibited minimum or no virus shedding.

**Table 1:** Virus detection in tracheal and cloacal swabs collected from vaccinated chickens after challenge with H5N1.

	1x vaccination^a^									2x vaccination									
	10			2			0.4			10			2			0.4			0
Chicken #	D2	D4	D7	D2	D4	D7	D2	D4	D7	D2	D4	D7	D2	D4	D7	D2	D4	D7	D2
1	−b	−	−	+	+	±†	−	−	−	−	−	−	−	−	−	−	−	−	+†
2	−	−	−	−	−	−	+	+	+†	−	−	±	−	−	−	−	+	+†	+†
3	−	−	−	−	−	−	+	−	−	−	−	−	±	−	−	−	−	−	+†
4	−	−	−	+	−	−	+	x†	x†	−	−	−	−	−	−	−	−	−	+†
5	−	−	−	−	−	−	−	−	−	−	−	−	−	−	−	−	−	−	+†
6	±	−	−	−	−	−	−	−	−	−	−	−	−	−	−	−	−	−	+†
7	−	−	−	−	−	−	−	−	−	−	−	−	−	−	−	+	+	−	+†
8	±	−	−	−	−	−	±	−	−	−	−	−	−	−	−	−	−	−	+†
9	−	−	−	−	±	−	+	+	+†	−	−	−	−	−	−	−	−	−	+†
10	−	−	−	−	−	−	+	x†	x†	−	−	−	−	±	−	−	−	−	+†

a Amount of sH5^3^ per immunization dose is indicated in µg. Day post infection (D) on which the tracheal and cloacal swabs were collected are indicated.

b + = positive; ± = inconclusive (low fluorescence [<0.07] after more than 31 cycles); − = negative; x = not tested.

† = chicken did not survive the challenge with HPAI H5N1.

### Efficacy of sH5^3^ vaccine against a lethal HPAI H5N1 infection in mice

**Figure 4:**
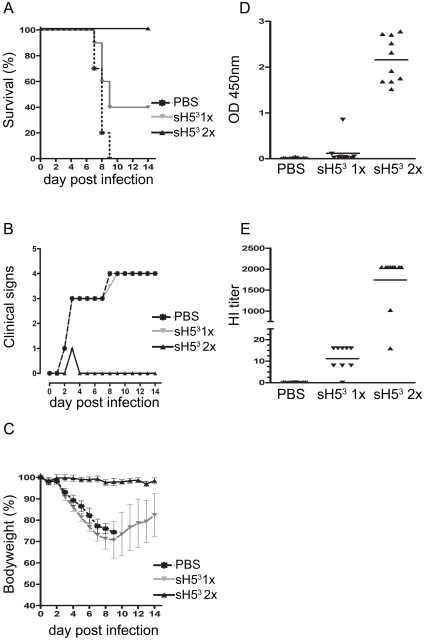
sH5^3^ vaccination of mice. Groups of 10 BALB/c mice were immunized i.m. with 2 µg sH5^3^ either once (day 21) or twice with a 3-week interval (day 0 and day 21). As a challenge control, one group of mice was mock-treated (PBS) twice (day 0 and day 21). Three weeks after the vaccination, mice were infected with ∼10 LD_50_ of A/Viet Nam/1194/04 and monitored daily for clinical signs and body weight during the next 14 days. (A) Kaplan-Meier survival curve indicating percentage mortality on each day for each group. (B) Median clinical scores per group. (C) Mean body weights per group expressed as percentage of starting body weight, plotted as a function of time. Error bars represent the standard deviation. (D–E) Blood samples were collected at the day of challenge. sH5^3^ antibody levels as determined by ELISA (D). HI titers against sH5^3^ (E). Bars represent geometric means.

Finally we examined whether sH5^3^ would also confer protection in mice. Therefore, 2 groups of 10 mice were vaccinated either once (on day 21) or twice (on day 0 and 21) with 2 µg of sH5^3^ adjuvanted with Stimune and challenged three weeks later by intranasal inoculation with ∼10 LD_50_ of H5N1 A/Viet Nam/1194/04. The percentage of mice surviving the infection, median clinical scores and body weights per group observed after the challenge inoculation are shown in Fig. 4. All mock-vaccinated mice succumbed to infection or had to be euthanized by day 9 p.i.. These mice showed severe clinical signs, including respiratory distress and significant weight loss, which continued until the animals died. A booster vaccination with sH5^3^ provided 100% protection against the lethal dose of A/Viet Nam/1194/04; none of the mice showed significant signs of disease and their body weights remained constant. A single vaccination with sH5^3^ did not protect mice against disease and concurrent weight loss; yet 40% of the mice survived. These mice started to recover from day 9 p.i. onwards as demonstrated by their regain of body weight. (Fig. 4C). Fig. 4D shows the pre-challenge anti-sH5^3^ antibody levels in individual serum samples,as determined by ELISA. Such antibodies could be detected in most vaccinated animals. However, after a single immunization, these levels remained very low compared to those in animals that received a booster vaccination. These results were essentially confirmed by determining the HI titers against sH5^3^ in the same serum samples. With the exception of one animal, the serum of which demonstrated high levels of auto-agglutination, all mice displayed high HI titers when vaccinated twice and low HI titers when vaccinated once. Thus, mice vaccinated twice with sH5^3^ were protected against a lethal challenge with HPAI H5N1, while a single vaccination provided partial protection. The differences in protection correlated with the observed serum HI titers.

## Discussion

Despite all the-understandable-attention drawn in the past year by media and scientific community to the new pandemic influenza A virus H1N1, a large influenza threat continues to be posed by HPAI H5N1. In 2009, HPAI H5N1 was found in wild birds in Germany, China, Mongolia and the Russion Federation, several outbreaks of the virus in poultry were reported in Viet Nam, Hong Kong, Nepal, India, Bangladesh, Egypt, Lao DPR, and Cambodia, while H5N1 remained endemic in large areas of Indonesia. That same year also dozens of confirmed human cases were reported (WHO timeline of major events; http://www.who.int/csr/disease/avian_influenza/ai_timeline/en/index.html). Even though the virus has so far remained restricted in its ability to infect humans and initiate efficient human-to-human transmission, its persistence and spread among wild birds and poultry holds a continual risk of the emergence of a pandemic strain. This threat can be reduced by vaccination of poultry against H5N1 as this would limit the propagation of the virus and minimize the risk of bird-to-human transmission. In addition, in case a human pandemic H5N1strain would emerge, there would be an immediate need for effective and reliable vaccines matching the pandemic strain. In the present study we evaluated the vaccine potential of a recombinant soluble H5 protein (sH5^3^) to protect chickens and mice against a lethal infection with HPAI H5N1. The recombinant HA vaccine, which has a short development cycle, proved to be protective after a single immunization in its natural host, while a booster vaccination was needed to confer complete protection in a mammalian model. In addition, the sH5^3^ induced immunity prevented viral shedding from chickens. These promising results warrant further research into the development of recombinant soluble HA as a fast, safe and effective alternative vaccine not only against H5N1, but against other influenza A viruses as well.

The recombinant HA expression cassette was constructed such that the HA protein was produced and secreted by cells in high yields as a bioactive trimer. The importance of the oligomeric state of the HA protein for the induction of neutralizing antibodies has recently been demonstrated [16]. High-molecular-weight oligomers and trimers, but not monomers, were found to efficiently induce neutralizing antibodies in mice. This difference was attributed to the preferential induction of antibodies against epitopes present in the monomeric, but not in the trimeric form [16]. While the soluble recombinant HA trimers were purified using metal affinity chromatography followed by ion-exchange chromatography in previous studies [16], [19], we used a protocol based on the Strep-tag system [23]. Proteins with a Strep-tag exhibit high affinity towards Strep-tactin, an engineered form of streptavidin. By exploiting this highly specific interaction, Strep-tagged proteins can be isolated in one step. Furthermore, because the Strep-tag elutes under gentle, physiological conditions it is especially suited for the generation of native proteins [24], a characteristic that in the case of HA may contribute to the ability of the recombinant protein to induce neutralizing antibodies.

The efficacy of the sH5^3^ vaccine was first studied in chickens. Adjuvanted with Stimune, a water-in-oil adjuvant also known as Specol, the sH5^3^ protein formulation induced an immunity that was completely protective against a lethal H5N1 challenge after administration of two doses (≥2 µg sH5^3^/dose). Importantly, our vaccine preparation also protected chickens after a single immunization. While 100% of the chickens were protected after vaccination with 10 µg sH5^3^, 90% were protected when using 2 µg. Similar HA doses were previously needed to protect chickens with a vaccine preparation consisting of full length HA proteins, which had been purified from insect cell cultures infected with recombinant baculovirus expressing the H5 gene, emulsified in a water-in-oil adjuvant [17]. These results are consistent with the observation that mammalian cell-produced HA trimers elicited similar levels of neutralizing antibodies as trimeric HA produced in insect cells [16]. The efficacy of our sH5^3^ vaccine preparation was furthermore demonstrated by the absence of viral RNA in the protected birds. This would imply that a vaccinated flock can pose a barrier against further spread of circulating virus.

Most conventional influenza vaccines require two vaccination rounds to produce antibody titers sufficiently high to confer full protection to chickens. In this regard, vaccination with sHA^3^ provides potential advantages over other vaccine approaches. However, the production costs per dose of sHA^3^ compared to egg-cultured inactivated whole-virus vaccines might be higher, even though recombinant protein expression in mammalian cell culture systems has been shown to be highly scalable and productive, with expression levels up to the order of grams of protein per liter [25], [26]. sHA^3^ vaccination could however be economically feasible in epidemic situations when millions of chickens have to be vaccinated individually, provided that a single preventive vaccination would suffice. Moreover, vaccinated flocks housed in endangered regions rapidly achieve a state of protective immunity. This may be a particularly valuable feature in the event of a pandemic, when the virus transmission cycle needs to be interrupted as soon as possible and the risk of exposure of farmers, veterinarians and people in monitoring teams to potentially zoonotic HPAI should be limited to the upmost extent.

The efficacy of the sH5^3^ vaccine was also studied in mice. The vaccine preparation was completely protective against a lethal H5N1 challenge after 2 doses. Immunization with a single dose resulted in 40% survival. These differences in protection levels correlated with the observed anti-sH5^3^ titers in the animals' sera. As the dose (2 µg) received by the mice is at least comparable, relative to their body weights, to the dose that conferred complete protection in chickens after a single immunization (10 µg), these results appear to suggest that the sH5^3^ is more effective in conferring a protective immune response in chickens than in mice. The reason for this apparent discrepancy is unclear and warrants further investigations. In the only other study so far that used soluble HA trimers as a vaccine preparation in mice, much less protection against challenge with H5N1 was observed after two immunizations [19]. Although the H5 trimer produced in this latter study differed from the one that we used, with respect to the trimerization motif (t4 foldon vs GCN4 trimerization motif) and the purification tag (His tag vs Strep tag II), the difference is more likely to be explained by the different adjuvant used (Alum vs Stimune). Alum is known to induce low antibody titers when used with subunit vaccines [27], while Stimune has been reported to generate long-lasting, functional antibody responses [28], [29], [30]. Stimune is however not licensed for human use.

In conclusion, we have shown that the sH5^3^ protein produced in mammalian cells elicited protective immune responses in mice and chickens when adjuvanted with Stimune. Chickens protected against the lethal H5N1 challenge also did not shed the virus at day 7 post infection. As these recombinant HA vaccines can be manufactured with high yields and a relatively short lead time, they offer an attractive alternative vaccination strategy, which will allow a rapid response to circulating and potentially pandemic influenza viruses.
